# Engaging today's undergraduate students in the field of dairy science with a focus on the female student population

**DOI:** 10.3168/jdsc.2024-0647

**Published:** 2024-09-18

**Authors:** Grace Lewis

**Affiliations:** Department of Animal and Food Science, University of Wisconsin–River Falls, River Falls, WI 54022

## Abstract

The dairy industry has profound economic importance, and individuals earning postsecondary degrees within dairy science–related fields are future industry leaders. Yet, enrollment at universities is declining and there is a labor shortage within the dairy industry. The objective of this review article is to highlight current trends within academia, identify some techniques to improve student experiences within higher education, and describe successful education strategies employed within dairy science–related fields. The proportion of female students within academia and agriculture-related degrees is growing; however, women still face gender bias and report higher incidence of mental health symptomology during their enrollment. With this, there is a profound need to address mental health–related issues throughout higher education, especially for the female student population. Additionally, active learning approaches and high-impact practices such as flipped classrooms, animal handling courses, capstone experiences, internships, and undergraduate research have shown promising benefits when employed in dairy and animal science–related fields. Universities play a critical role in guiding the future leaders of the dairy industry into successful and fulfilling careers, and this review highlights some pertinent strategies to improve the engagement of dairy science undergraduate students.

## Highlights

• Academia faces declining enrollment and increased mental health-related concerns.
• Mental health is a barrier for many students and needs to be properly addressed.
• Active learning approaches have shown promise in dairy science-related fields.
• High-impact practices (e.g., internships) benefit dairy science-related programs.

## Graphical Abstract

**Figure:**
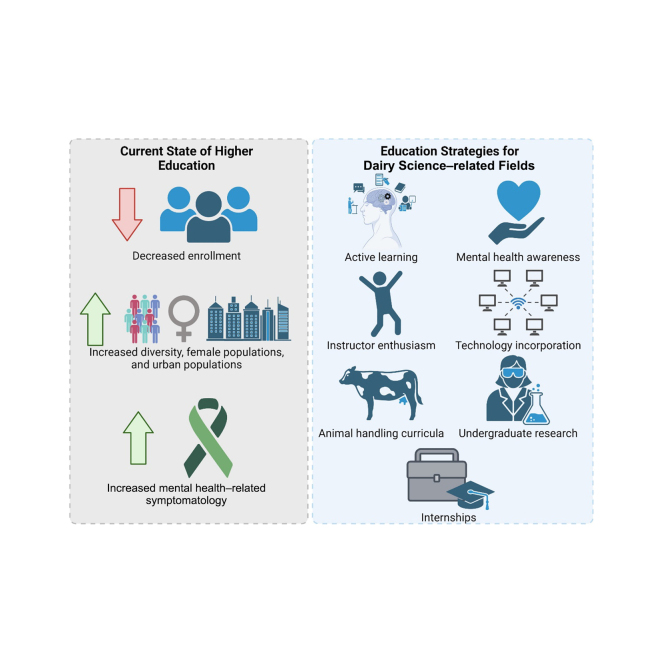
**Summary:** Individuals studying dairy science-related fields will function as the future of the dairy industry. However, dairy industry trends reflect labor shortages and a declining workforce, and higher education is facing declining enrollment and demographic shifts. With this, there is a need to implement support programs and teaching strategies that improve student experiences within these areas of study. Specifically, mental health-related concerns are a major barrier to student enrollment and retention, especially for female students, and these factors must be addressed within higher education. Many pedagogical strategies can improve student experiences in dairy science-related fields, including instructor enthusiasm, active learning, and high-impact practices (e.g., undergraduate research and internships). Together, these approaches should be considered to mitigate concerning trends and propel the dairy industry into the future. Figure created with BioRender.com.

The dairy industry in the United States generates $42 billion in direct wages and supplies 3.3 million jobs (Gibeson, 2023). However, like other industries, the dairy industry is currently facing labor shortages with 64% of dairy company chief executive officers listing labor shortages among their primary concerns (Grady, 2024). These labor shortages result in reduced hours of operation, higher farm wages, increased dairy product costs, and reliance on foreign production (Rutledge, 2023).

Individuals earning advanced degrees in dairy science–related fields are the future leaders of the dairy industry, and there are currently >50 dairy science 4-yr undergraduate programs within the United States. Numerous demographic changes have been identified within agriculture-related undergraduate programs including greater proportions of female students, nontraditional students (e.g., older students), and urban students than before (Buchanan, 2008). Additionally, fewer of the students in these programs desire to return to their family farming operation. Although many land-grant universities have more women studying agriculture than men, the proportion of women in leadership roles throughout the dairy industry is lacking with only 4.1% of dairies in the United States having a female principal operator (Allen, 2023). Improving the success of these various dairy science programs is critical to guiding the dairy industry into the future, and there should be focused and targeted efforts to inspire women in these career paths.

Regardless of the need for individuals earning advanced degrees in dairy science– and agriculture-related fields, the current state of higher education shows concerning trends with enrollment numbers declining since 2010 (Table 1; National Center for Education Statistics, 2022). According to The State of Higher Education Survey 2024, among adults without a college degree, 75% of individuals agreed that bachelor's degrees are extremely or very valuable (Gallup and Lumina Foundation, 2024). Despite this, enrollment at these higher education institutions is declining, with cost identified as the greatest barrier to enrollment, especially for women. However, even after enrollment, student retention is a primary concern. Specifically, among currently enrolled students participating in the same survey, 32% of students reported that they considered withdrawing from their bachelor's degree program, and these percentages were even higher among female students (i.e., 38%; Gallup and Lumina Foundation, 2024). The leading reasons students considered withdrawing from their university were mental and emotional health–related concerns.

**Table 1:** Total fall enrollment in degree-granting postsecondary institutions from 2000 to 2022, modified from the US Department of Education^1^

Year	Total enrollment	Sex of student		
		Male	Female	Percent female
2000	15,312,289	6,721,769	8,590,520	56.1
2001	15,927,987	6,960,815	8,967,172	56.3
2002	16,611,711	7,202,116	9,409,595	56.6
2003	16,911,481	7,260,264	9,651,217	57.1
2004	17,272,044	7,387,262	9,884,782	57.2
2005	17,487,475	7,455,925	10,031,550	57.4
2006	17,754,230	7,572,265	10,181,965	57.3
2007	18,258,138	7,819,938	10,438,200	57.2
2008	19,081,686	8,177,714	10,903,972	57.1
2009	20,313,594	8,732,953	11,580,641	57.0
2010	**21,019,438**	**9,045,759**	11,973,679	57.0
2011	21,010,590	9,034,256	**11,976,334**	57.0
2012	20,644,478	8,919,006	11,725,472	56.8
2013	20,376,677	8,861,197	11,515,480	56.5
2014	20,209,092	8,797,530	11,411,562	56.5
2015	19,988,204	8,723,819	11,264,385	56.4
2016	19,846,904	8,638,422	11,208,482	56.5
2017	19,778,151	8,571,314	11,206,837	56.7
2018	19,651,412	8,444,614	11,206,798	57.0
2019	19,630,178	8,363,889	11,266,289	57.4
2020	19,027,410	7,885,033	11,142,377	**58.6**
2021	18,658,756	7,767,866	10,890,890	58.4
2022	18,580,026	7,814,536	10,765,490	57.9

1 Highest values for each column are bolded. National Center for Education Statistics, 2022.

Considering this, the objective of this review is to highlight some strategies used to promote student recruitment, retention, and persistence within the field of dairy science with an emphasis on practices that target female student populations. The present work should help to identify strategies to optimize learning experiences for students in higher education.

Although the goal of this work is to target dairy science specifically, there are limited articles related to undergraduate teaching in this specific field. Therefore, it was necessary to expand the scope of this literature review to closely related fields (e.g., animal science, veterinary medicine). The references from more general majors were minimally cited, and these citations are clearly designated within the article. Statistics on higher education within the United States were collected primarily from The State of Higher Education 2024 survey (Gallup and Lumina Foundation, 2024), and these values were not specific to dairy science–related fields or one specific institution. Due to educational system differences, the literature included within this review is from the United States with few, clearly designated exceptions.

As highlighted above, the primary reason enrolled students consider dropping out from a higher education program is due to *mental health–related concerns* (Gallup and Lumina Foundation, 2024). In 2021, of the 18.5 million Americans attending college, 37% and 44% of students report symptoms of anxiety and depression, respectively (Kreuziger and Snethen, 2023). The percentage of students experiencing mental health–related symptoms was growing before the COVID pandemic, but the pandemic expedited this trend.

In The State of Higher Education 2024 survey, of 3,005 surveyed individuals not currently enrolled in higher education, >50% of individuals under 35 yr old considered mental health or emotional stress as a very important reason preventing enrollment (Gallup and Lumina Foundation, 2024). When enrolled, many students still did not feel emotionally supported by their institution, especially minority groups. Concerningly, only 67% of female students involved in this survey felt that they “belonged at their institution” compared with 77% of male students. Of these enrolled students (n = 6,015), 22% have considered dropping out of their programs due to mental or emotional health challenges with even higher numbers reported for female students (i.e., 28%).

There is clear discrepancy between the reported occurrence of mental health–related concerns for female students versus male students in higher education with women more likely to report anxiety, trauma, and depressive symptomology, self-harming behavior, suicide attempts, and other mental health–related concerns (Anastasiades et al., 2017; Reidy and Wood, 2024). Programs that consider these concerns as “institutionally centered” (i.e., attributed to factors beyond personal characteristics such as patterns of exclusion or varying expectations) instead of “student-centered” (i.e., related to the intrinsic characteristics of female students such as levels of confidence or motivation) are likely to make more progress on the inclusion and mental health of their female student population (Fox et al., 2009). In fact, some studies report that, despite outnumbering and outperforming their male peers, female students are still contending with gender bias in some scientific fields (Bloodhart et al., 2020). Furthermore, undergraduate women in gender-balanced science, technology, engineering, and mathematics (**STEM**) disciplines reported more sexual violence (e.g., sexual coercion, rape) than their counterparts in non-STEM majors. These studies highlight the “institutionally centered” nature of these issues, and institutions should make a conscious effort to protect, support, and inspire their female student population.

Although these data are not specific to dairy science–related fields, mental and emotional support for university students is a starting point for all programs, and enrollment cannot be expected to improve until these primary needs are met. In a systematic review of college instructors' experiences with undergraduate student mental health symptoms, Kreuziger and Snethen (2023) highlighted an opportunity to provide more mental health education for faculty as well as a need to make mental health–related policies and laws easily accessible. Although faculty-student interactions should not be confused with counselor-patient relationships, there is a supreme need to provide instructors the necessary training to handle mental health–associated situations as they are so prevalent throughout the undergraduate student demographic.

A student's perception of an academic course can be greatly influenced by the instructor's attitude and the technique used to convey information. However, college faculty are often hired for their technical or scientific expertise, and their teaching ability is assumed to be less of a priority. The success of an academic class is reliant on the instructor, and there should be heightened focus on the pedagogical skills of faculty members with focused attempts to provide opportunities for faculty to develop these skills. For example, it is critical for programs to encourage students to be engaged within the classroom, and a primary source of this encouragement is *instructor enthusiasm*. When students perceive their instructor to be enthusiastic, they are more likely to persist through their degrees and into a degree-related career (Yan et al., 2023). In a Chinese study of 358 first- and third-year college students, Yan et al. (2023) found a significant, direct correlation between perceived teacher enthusiasm (i.e., high ratings on questions such as: “Our class teacher teaches with passion”) and learning engagement (i.e., the students' efforts in learning activities). Furthermore, learning engagement was also significantly correlated with professional commitment (i.e., the individual's dedication to the profession). There were no studies evaluating the relationship between teacher enthusiasm and professional commitment conducted within the United States.

Many teaching approaches have been shown to benefit student learning. The primary teaching approach that will be discussed in this article is *active learning*. Active learning differs from a traditional lecture-style format as it is learner-centered and relies on student engagement with the learning process (Ragland et al., 2023). Active learning is an umbrella term that can take many forms, and some examples include case studies, flipped classrooms, think-pair-share, polling questions, discussions, and critical reflections. There are also examples of active learning that are commonly employed in dairy and animal science curricula such as animal handling courses, internships, and undergraduate research, and the application of these various experiences in coursework will be discussed below.

The goal of active learning is to increase student engagement as it requires students to drive their own progress (Ragland et al., 2023). In practice, active learning creates lifelong learners by focusing on the learning process instead of the products of learning. Even with the benefits of this approach, a study conducted by Ferree et al. (2022) found a preference for a lecture-based teaching approach compared with a case-based teaching approach (i.e., materials presented as a case study) for 25 students enrolled in an undergraduate dairy cattle management class pursuing a degree in animal science. This study highlights one of the downfalls of this approach as some students prefer easily accessible information instead of information that must be obtained through application, analysis, and synthesis. With that, a combination of lecture and active learning styles might be more successful in some academic settings. Team-based learning, which involves the organization of students into permanent collaborative teams within a larger academic class, can be an appropriate active learning style for larger classes (Ng and Newpher, 2020).

Another active learning approach is the *flipped classroom*, which involves students watching online lectures in their own time to prepare for interactive class activities during their scheduled lecture time (Mortensen and Nicholson, 2015). The flipped classroom approach targets students who may have a decreased tolerance for a typical lecture-based style and are familiar with accessing information with technological tools. Although an alternative teaching style like a flipped classroom can be unfamiliar to students, there are studies that reflect benefits to this approach. In a study conducted by Mortensen and Nicholson (2015), students enrolled in an Introduction to Equine Science course were taught using a flipped classroom format (n = 130) and compared with previous students taught in a more traditional, lecture-style format (n = 173). In the flipped classroom format, students viewed prerecorded video lectures and participated in reflection assignments, a semester project with weekly discussion board posts, and educational games. Students taught in the flipped format scored significantly higher on all 3 exams throughout the semester (*P* < 0.05) and indicated more positive learning experiences than the students taught using the traditional style. A flipped classroom format can provide students the freedom to actively engage with course material and apply more critical thinking skills.

With the modern reliance on technology in every component of life, incorporating *technology-based activities* into undergraduate teaching curricula in place of traditional discussion has shown promise for some programs. Whittaker et al. (2014) incorporated the use of a student-friendly social networking site (i.e., Facebook) to replace traditional discussion boards as part of an undergraduate animal science class (n = 44). Using this format, the researchers were able to develop an online learning community with rapid response rates, positive social interactions, and heightened student engagement. Beyond social media, other modern technologies, such as artificial intelligence and machine learning, are being applied within the dairy industry (De Vries et al., 2023), and it is beneficial to introduce students to these tools during their undergraduate career. Using technology, distance-based learning has also been successfully used in animal science–related courses (Bing et al., 2011).

Beyond teaching approaches, there are also specific academic experiences that have been shown to benefit student learning in dairy science–related fields. The specific curricula that will be discussed in this article include animal handling courses, capstone courses, internships, and undergraduate research; all of these are examples of *high-impact practices*.

To start, in animal science–related courses, having an academic course that incorporates *animal handling experiences* is critical to prepare these students for their future careers. In a study conducted by Bundy et al. (2019), the majority of students enrolled in an Introductory Animal Handling course (n = 87) felt that hands-on learning experiences were beneficial for learning. Furthermore, the background of these students (e.g., farming experience) did not influence post-exam scores despite their influence on pre-exam scores, reflecting that animal handling courses can equalize students' familiarities with animal handling regardless of the students' prior experiences. Incorporating these courses into academic curricula is critical as more students in agriculture-related majors are from urban backgrounds.

Similarly, *capstone courses*, *internships*, and *undergraduate research* programs have demonstrated benefits in dairy and animal science programs. A capstone experience, defined as a high-impact educational practice that requires students to apply the culmination of their degree knowledge, can include internships, undergraduate research, study abroad programs, and independent studies. A study conducted by Hall et al. (2017) reported that 81% of animal and poultry science students (n = 92 alumni and 4 current students) who participated in a capstone experience felt more prepared for their career goals after its completion. In a separate study, Peffer (2012) surveyed 734 animal science students who completed an internship program and found that 94% of students found their internship to be a valuable experience. Cresiski et al. (2021) evaluated the influence of undergraduate research on students enrolled at a teaching-oriented college (n = 1,472) and found that students involved in undergraduate research were significantly (*P* < 0.01) more likely to persist and graduate from their university compared with students who did not participate in undergraduate research. Note, this study was not specific to students within dairy science–related fields. For larger programs with limited undergraduate research opportunities, undergraduate research can be incorporated directly into animal science programs as demonstrated by Jones and Lerner (2019).

There are also many dairy science–related collegiate experiences that incorporate high-impact practices outside of the classroom including the North American Intercollegiate Dairy Challenge, National Collegiate Dairy Judging Contest, Collegiate Dairy Products Evaluation Contest, Dairy Management Inc. New Dairy Product Competition, Animal Welfare Assessment Contest, and others. International and national conferences (e.g., American Dairy Science Association Annual Meeting, World Dairy Expo) also incorporate undergraduate-specific activities and events, providing additional engagement opportunities for students.

Capstone and other industry-related experiences allow students to apply their knowledge into projects with tangible and practical outcomes, allowing students to experience the real-world impact of their education. Even with the incorporation of real-world experiences into coursework, students may need additional guidance to find a fitting career pathway. Career exploration is an important practice that allows students to identify and analyze personal factors (e.g., passions) and external factors (e.g., salary) to guide them to make informed decisions about their desired profession. This exploration can be accomplished through a variety of means including personality tests, professional networking, online career counseling, virtual career games, and many more. Many studies have demonstrated the benefits of these practices such as facilitated career-related decision making, enhanced self-awareness and confidence, and better developed career-related capabilities and resources (Jiang et al., 2019). Importantly, career exploration is a dynamic practice that can be adapted throughout career stages.

In summary, the goal of this review was to highlight some concerning trends within higher education and provide approaches to consider for the mitigation of these issues. Although most of these approaches can be applied to any advanced degree program, the majority of the cited references emphasize successful approaches within dairy and animal science–related fields. It is necessary to highlight mental and emotional health–related concerns as a major barrier for students pursuing advanced degrees, especially for female students. There is a profound need to address these issues within universities. Students who are engaged within the classroom are more likely to persist within their respective fields, and this engagement can be encouraged through a variety of techniques such as enthusiastic teaching, active learning approaches, high-impact practices, and career exploration. Universities should monitor student involvement in high-impact practices and invest in the opportunities that drive student engagement. Furthermore, there is a need for well-conducted and publishable studies on novel teaching approaches within the dairy science field, especially studies that connect undergraduate teaching styles and high-impact practices to post-graduation commitment to the profession. With this approach, the strategies used within academic curricula can be related to employment within a dairy science–related career, and this relationship can work to reduce workforce shortages and drive the dairy industry into the future.
